# Extracorporeal membrane oxygenation in critical airway interventional therapy: A review

**DOI:** 10.3389/fonc.2023.1098594

**Published:** 2023-03-27

**Authors:** Hongxia Wu, Kaiquan Zhuo, Deyun Cheng

**Affiliations:** ^1^ Department of Respiratory and Critical Care Medicine, West China Hospital, Sichuan University, Chengdu, China; ^2^ Department of Neurosurgery, Suining Municipal Hospital of Traditional Chinese Medicine (TCM), Suining, China

**Keywords:** extracorporeal membrane oxygenation, interventional therapy, bronchoscopy, malignant tumor, airway stenosis, airway obstruction

## Abstract

**Introduction:**

Extracorporeal membrane oxygenation (ECMO) is widely used during refractory cardiac or respiratory failure, and some case reports described ECMO utilization in critical airway interventional therapy.

**Methods:**

Eligible reports about patients receiving airway interventional therapy under ECMO were retrieved from Web of Science, Embase, Medline, and Cochrane databases up to 1 August 2022.

**Results:**

Forty-eight publications including 107 patients who underwent ECMO for critical airway problems met the inclusion criteria. The critical airway problem that was reported the most was tumor-associated airway obstruction (n = 66, 61.7%). The second most reported etiology was postoperative airway collapse or stenosis (n = 19, 17.8%). The main interventional therapies applied were airway stent placement or removal (n = 61, 57.0%), mass removal (n = 22, 20.6%), and endotracheal intubation (n = 12, 11.2%) by bronchoscopy. The median ECMO duration was 39.5 hours. Eleven patients had ECMO-associated complications, including seven cases of airway hemorrhage, one case of arteriovenous fistula, one case of vein rupture and hematoma, one case of foot ischemia, and one case of neuropraxia of the cannulation site. In total, 91.6% of the patients survived and were discharged from the hospital.

**Conclusion:**

ECMO appears to be a viable form of life support for patients undergoing interventional therapy for critical airway problems.

## 1 Introduction

Critical airway problems include airway obstruction, massive airway hemorrhage, and acute tracheal or bronchial lesions, which may lead to severe hypoxia or death. Airway obstruction is the leading cause of critical airway problems. Respiratory interventional therapies, including airway stent placement and mass removal by bronchoscopy, are effective in treating airway problems (1, 2). Most of these procedures can be safely and successfully performed using various ventilation techniques (3). However, interventional therapy can be particularly dangerous and cause hypoxemia in patients with critical airway problems such as near-total airway obstruction.

Extracorporeal membrane oxygenation (ECMO) has become an important intervention for patients with severe cardiac or pulmonary dysfunction for whom conventional therapies fail (4). The expanded utilization of ECMO in non-traditional indications, such as upper airway surgery and malignant airway obstruction, has been reported in some studies. Critical airway problems indicate that ECMO prevents or overcomes deadly hypoxemia and hypercapnia. This review summarizes the reports on the combination of ECMO and airway interventional therapy for critical airway problems.

## 2 Materials and methods

### 2.1 Search strategy

We searched the Cochrane, Web of Science, Medline, and Embase databases for studies using the terms “{[extracorporeal membrane oxygenation (Title/Abstract)] OR [extracorporeal life support (Title/Abstract)]} AND {[airway (Title/Abstract)] OR [trachea (Title/Abstract)] OR [bronchial (Title/Abstract)]}”. We searched for reports from database inception until 1 August 2022. We also retrieved the references and keywords of all included studies to reduce bias. The results were according to the Preferred Reporting Items for Systematic Reviews and Meta-Analyses guidelines. This study was registered with PROSPERO (No. CRD42022367887).

### 2.2 Inclusion and exclusion criteria

The inclusion criteria were as follows: 1) randomized controlled trials, single-arm cohort studies, or case reports; 2) trials conducted in patients with critical airway problems; and 3) trials reporting data on ECMO and interventional therapy. Patients were excluded if they were admitted for other medical problems or if they had been diagnosed with brain death.

### 2.3 Study selection

Two investigators performed study selection and recorded the reasons for exclusion. A third reviewer was consulted if no consensus was reached.

### 2.4 Data extraction

Two investigators independently extracted and recorded the study’s demographic characteristics and outcomes in a standard form, as recommended by Cochrane. A third investigator resolved any disagreements.

### 2.5 Results

Outcome measures were the underlying etiology of critical airway problems, ECMO-associated complications, anticoagulation therapy, interventional therapy, and in-hospital survival rate.

### 2.6 Statistical analysis

Data are presented as the median and interquartile range (IQR) or absolute numbers and percentages. Data were analyzed using SPSS version 25.0 (IBM, Armonk, NY, USA).

## 3 Results

### 3.1 Characteristics of clinical trials

**Figure 1:**
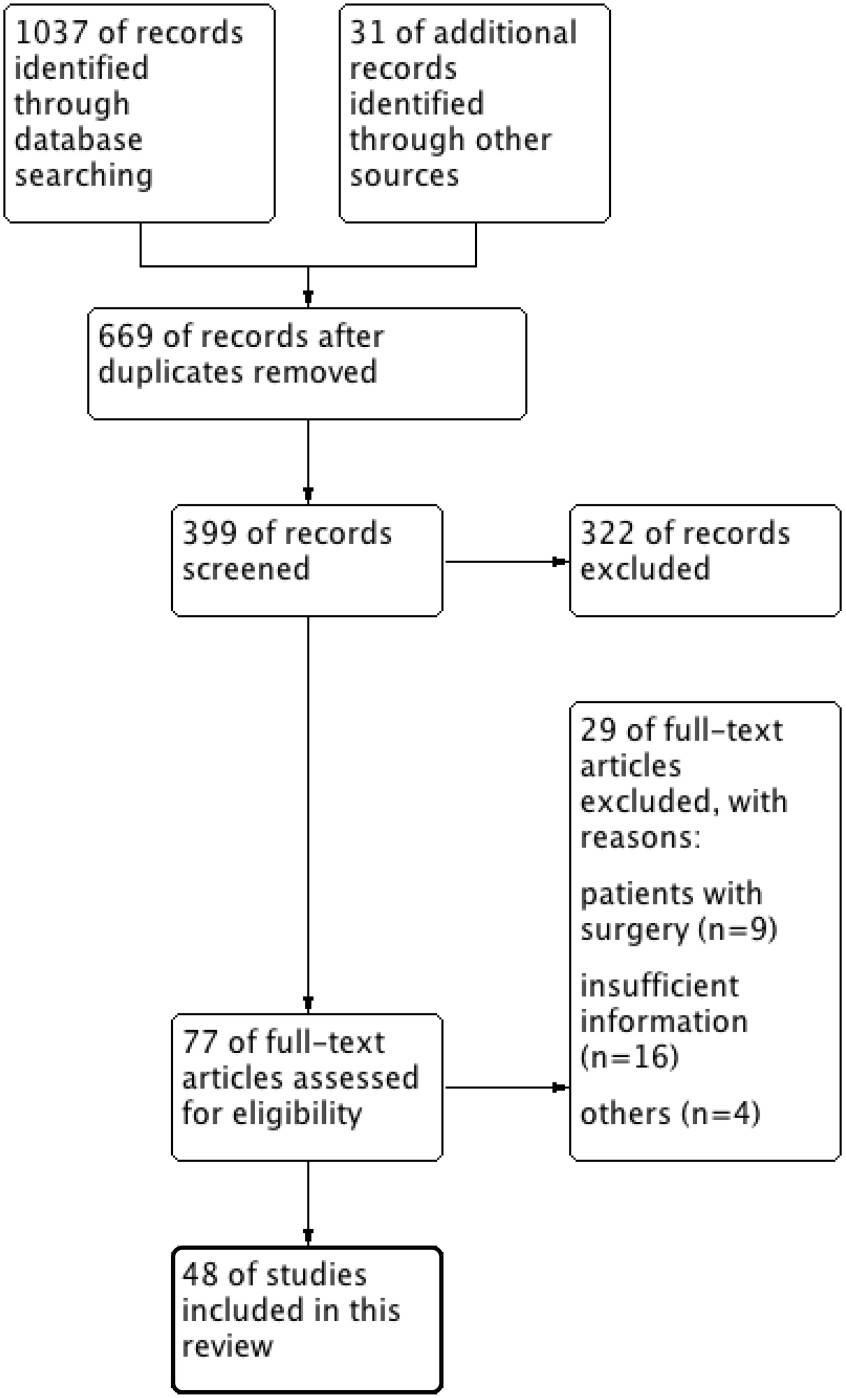
Flow diagram of selected searches for inclusion in this review.

Forty-eight publications (5–52) including 107 patients who underwent ECMO for critical airway problems met the inclusion criteria. One patient underwent ECMO twice. Details of the study selection process are presented in **Figure 1**. All reports were case reports or case series. The median age was 57 (IQR 40, 67) years, and men accounted for 61.7% (n = 66) of the patients. Emergent ECMO accounted for 53.3% (n = 57) of the cases. Details of the included studies are presented in **Supplementary Tables 1**, **2**.

### 3.2 Main outcomes

#### 3.2.1 Underlying etiology

Most of the critical airway problems were tumor-associated airway obstructions (n = 66, 61.7%), among which malignant tumor invasion or metastasis and benign tumor occlusion or oppression accounted for 57.0% (n = 61) and 4.7% (n = 5), respectively. The second most reported etiology was postoperative airway collapse or stenosis (n = 19, 17.8%). Congenital or idiopathic airway stenosis (n = 8, 7.5%), foreign body aspiration (n = 7, 6.5%), airway hemorrhage (n = 4, 3.7%), inflammatory disease (n = 4, 3.7%), migration or fracture of airway stent (n = 3, 3.7%), nearly tissue or organ oppression (n = 2, 1.9%), and airway lesions or fistulas (n = 2, 1.9%) were the other indications. Lung cancer (n = 27, 25.2%) was the most common indication of airway obstruction in our review. The details are presented in **Table 1**.

**Table 1:** Underlying etiologies and survival rate of patients with critical airway problems receiving ECMO.

Underlying etiologies*	No. (%)	Survival rate (%)
**Tumor-associated airway obstruction**	66 (61.7)	90.5
**Malignant tumor invasion or related metastasis**	61 (57.0)	90.0
Lung cancer	27 (25.2)	
Thymus cancer	1 (0.9)	
Tracheal cancer	4 (3.7)	
Thyroid cancer	4 (3.7)	
Lymphoma	8 (7.5)	
Esophageal cancer	6 (5.6)	
Malignant melanoma	2 (1.9)	
Gastric cancer	1(0.9)	
Head and neck cancer	1 (0.9)	
Renal cell carcinoma	1 (0.9)	
Osteogenic sarcoma/chondrosarcoma	2 (1.9)	
Schwannoma	1 (0.9)	
Myeloma	1 (0.9)	
Testicular cancer	1 (0.9)	
Adenoid cystic carcinoma	1 (0.9)	
**Benign tumor occlusion or oppression**	5 (4.7)	100
Recurrent tracheal papillomatosis	3 (2.8)	
Mediastinal teratoma	1 (0.9)	
Neurofibromatosis	1 (0.9)	
**Postoperative airway collapse or stenosis**	19 (17.8)	100
Post-tracheostomy	4 (3.7)	
Post-intubation	10 (9.3)	
Post-lung transplant	2 (1.9)	
Post-pneumonectomy	3 (2.8)	
**Nearly tissue or organ oppression**	2 (1.9)	100
Thoracic aortic aneurysm	1 (0.9)	
Adenomatous goiter	1 (0.9)	
Foreign body aspiration	7 (6.5)	100
**Congenital or idiopathic airway stenosis**	8 (7.5)	87.5
**Migration or fracture of airway stent**	4 (3.7)	100
**Airway hemorrhage**	4 (3.7)	75.0
**Inflammatory disease**	4 (3.7)	100
**Airway lesion or fistula**	2 (1.9)	100

No., number; ECMO, extracorporeal membrane oxygenation.

*Some patients had more than one ECMO etiology.

#### 3.2.2 Interventional therapy

The main interventional therapies were airway stent placement, replacement, or removal by bronchoscopy (n = 61, 57.0%), mass removal (n = 22, 20.6%), and endotracheal intubation (n = 12, 11.2%) by bronchoscopy. Other therapies included biopsy (n = 6, 5.6%), tracheostomy (n = 5, 4.7%), foreign body extraction (n = 7, 6.5%), laser (n = 4, 3.7%), suction and removal of blood clots (n = 3, 2.8%), balloon dilatation (n = 3, 2.8%), bronchoalveolar lavage (n = 2, 1.9%), endobronchial balloon occlusion and hemostatic therapy (n = 1, 0.9%), cryotherapy (n = 1, 0.9%), and photodynamic therapy (n = 1, 0.9%). Some patients received several adjunctive therapies. The details are presented in **Table 2**.

**Table 2:** Interventional therapies of patients with critical airway problems receiving ECMO.

Interventional therapies*	No. (%)
Placement, removal, or replacement of airway stent	61 (57.0)
Mass removal	22 (20.6)
Extraction of foreign body	7 (6.5)
Suction and removal of blood clots	3 (2.8)
Biopsy	6 (5.6)
Bronchoalveolar lavage	2 (1.9)
Endotracheal intubation	12 (11.2)
Tracheostomy	5 (4.7)
Balloon dilatation	3 (2.8)
Laser therapy	4 (3.7)
Endobronchial balloon occlusion and hemostatic therapy	1 (0.9)
Cryotherapy	1 (0.9)
Photodynamic therapy	1 (0.9)

No., number; ECMO, extracorporeal membrane oxygenation.

*Some patients received more than one interventional therapy.

#### 3.2.3 ECMO-associated complications and anticoagulation therapy

In this review, 89 patients who received venovenous-ECMO (VV-ECMO) for refractory hypoxia or hypercapnia were included. In comparison, 18 patients received venoarterial-ECMO (VA-ECMO) for hemodynamic instability or cardiopulmonary resuscitation. The ECMO cannulation sites were as follows: both femoral veins (n = 39), femoral and internal jugular veins (n = 30), femoral vein and artery (n = 12), single cannulation with a double lumen in the internal jugular vein (n = 4), internal jugular vein and artery (n = 1), and not mentioned (n = 21). The median total ECMO duration was 39.5 (IQR 17.4, 100.8) hours. Unfractionated heparin (n = 59) was the most commonly used anticoagulant. In 13 patients, continuous infusion of nafamostat mesylate and heparin was administered to maintain the targeted activated clotting time (ACT). Two patients did not receive anticoagulation therapy because of the high risk of bleeding. ACT was used as a monitor for anticoagulation therapy in most studies. The range of ACT was 130–250 seconds in most studies. Some reports did not record the exact anticoagulation therapy used. Only one patient was not successfully weaned off ECMO for the underlying disease. Eleven patients reported ECMO-associated complications, including seven cases of airway hemorrhage, one case of arteriovenous fistula, one case of vein rupture and hematoma, one case of foot ischemia, and one case of neuropraxia at the cannulation site. One patient died of massive airway bleeding after being weaned off ECMO. The details are presented in **Table 3** and **Supplementary Table 2**.

**Table 3:** Survival rate of patients with different ECMO-associated complications.

Complications	Total	Death	Survival rate (%)
Foot ischemia	1	0	100
Massive airway bleeding	7	1	85.7
Vein rupture and hematoma	1	0	100
Arteriovenous fistula	1	0	100
Neuropraxia of the cannulation site	1	0	100
Total	11	1	90.9

ECMO, extracorporeal membrane oxygenation.

#### 3.2.4 Survival rate

In total, 91.6% (n = 98) of the patients survived and were discharged from the hospital. Among the nine patients who died, eight died of the underlying disease, and only one died of ECMO-associated massive airway hemorrhage. During the follow-up period of 60 days in 53 patients, seven died due to the progression of the underlying disease. The details are presented in **Table 4** and **Supplementary Table 1**.

**Table 4:** Survival rate of patients with different ECMO indications or modes.

ECMO indications* or modes	Total	Death	Survival rate (%)
Malignant tumor invasion or related metastasis	60	7	88.3
Benign tumor occlusion or oppression	3	0	100
Postoperative airway collapse or stenosis	19	0	100
Nearly tissue or organ oppression	2	0	100
Foreign body aspiration	7	0	100
Congenital or idiopathic airway stenosis	8	1	87.5
Migration or fracture of airway stent	4	0	100
Airway hemorrhage	4	1	75.0
Inflammatory disease	4	0	100
Airway lesion or fistula	2	0	100
VA-ECMO	18	1	94.4
VV-ECMO	90^#^	8	91.0

VA, venoarterial; VV, venovenous; ECMO, extracorporeal membrane oxygenation.

*Some patients had more than one ECMO indication.

^#^One patient underwent VV-ECMO twice.

## 4 Discussion

In our review, airway obstruction caused by malignant tumors was found to be the primary cause of critical airway problems. Surgical resection and reconstruction may provide the highest probability of definitively managing malignant tumors. However, most patients with malignant airway obstruction cannot undergo surgery for metastatic disease or are in poor physical condition (53). Respiratory interventional therapy in patients with acute severe airway problems provides adequate emergency ventilation, palliation, and airway stabilization, allowing for additional treatments. Bronchoscopy plays a beneficial role in treating patients with malignant airway obstruction (54, 55). However, bleeding may be the most common complication of interventional therapy.

In critically ill patients, interventional airway therapy can be hazardous and cause life-threatening hypoxemia. Additionally, endotracheal intubation for mechanical ventilation may be impossible or even fatal. VV-ECMO is used for treating critical respiratory failure, and VA-ECMO provides both respiratory and cardiac support (56). To date, a series of studies have reported a combination of ECMO and interventional therapy for critical airway problems. However, the available evidence is only based on case reports and case series studies.

This review analyzed published reports on ECMO-assisted respiratory interventional therapy in patients with critical airway problems. VV-ECMO provides adequate oxygenation during surgical or bronchoscopic interventions, and VA-ECMO should be used in high-risk patients with hemodynamic instability (16). Serino et al. reported that lung cancer accounted for 57.1% of patients with malignant airway obstruction (n = 56) and many other cancers through metastasis (57). ECMO for critical airway problems has been initially reported in pediatric populations and has been successfully used in interventional therapy in children for foreign body aspiration (11, 12, 25), congenital tracheal stenosis (6), and endotracheal masses (14). In adults, the first use of ECMO in a patient with sawdust aspiration undergoing foreign body extraction by bronchoscopy was published by Higashi in 1989 (5). For malignant central airway occlusions, airway stenting is an optional therapy (58, 59). As a palliative therapy, stenting can relieve symptoms immediately and improve quality of life (60). Extrinsic or endobronchial obstruction and tracheoesophageal fistula are indications for stenting in malignant tumors (61). In our review, airway stent placement *via* bronchoscopy was the most commonly used interventional therapy. However, complications associated with stent placement have also been reported. Serino et al. (57) reported that 37.5% of patients with stent placement were associated with ≥1 complication, including mucositis, migration, tumor in-growth, and granulation tissue. However, no case of airway hemorrhage during interventional therapy has been reported.

Various complications associated with ECMO have been reported in critically ill patients (4). These complications may affect patient outcomes and mortality rates. The most common complications associated with VV-ECMO are bleeding and venous thrombosis (62). In our review, airway bleeding was found to be the most common complication. No post-cannulation venous thrombosis was reported because of the relatively short duration of ECMO. Limb ischemia is a common complication of VA-ECMO. However, distal limb reperfusion with an antegrade catheter markedly reduces its incidence (63).

Most patients included in this review experienced immediate relief from symptoms and survived, suggesting that ECMO is practical and safe during interventional therapy in patients at high risk of airway blockade. This study is the first comprehensive analysis of the efficacy of ECMO in critical airway interventional therapy in a large number of patients. Nevertheless, the following limitations should be considered. First, all included studies were case or case series, and the application of ECMO for critical airway therapy is relatively rare. However, avoiding ECMO therapy in patients with a high risk of airway obstruction or refractory cardiac or pulmonary failure does not conform to ethical standards. Second, the patients had different underlying diseases that influenced their survival rates. Third, anticoagulation therapy and targeted ACT differed between studies, which may have affected the incidence of bleeding. Nevertheless, the results of this study have specific value and significance, and further research is warranted.

## 5 Conclusion

In conclusion, ECMO is viable for providing adequate cardiac or respiratory support, with an acceptable risk of complications. ECMO accompanied by airway interventional therapy can be successfully performed in patients with critical airway problems. However, further studies are needed to validate these standardized procedures.

## Author contributions

HW initiated and coordinated the study. KZ and HW were responsible for the data collection and data analysis. Studies were reviewed by DC. HW wrote the first draft of the manuscript. All authors contributed to the article and approved the submitted version.
