# Supplementary material for: Extracorporeal membrane oxygenation in critical airway interventional therapy: A review
Source: Front Oncol. 2023 Mar 27;13:1098594. doi: 10.3389/fonc.2023.1098594 (PMC10083385; doi:10.3389/fonc.2023.1098594)
Supplement: Supplementary file 1 [file Table_1.docx]

| First Author | Year | Region | No. | Gender | Age，  years | Causes of Airway Obstruction | Obstruction Site | Interventions | ICU stay, days | Hospital stay, days | Outcomes | Survival  ＞60 days |
| --- | --- | --- | --- | --- | --- | --- | --- | --- | --- | --- | --- | --- |
| Higashi | 1989 | Japan | 1 | M | 17 | Sawdust aspiration | T, LMB, RMB | Foreign body extraction | NM | NM | Survival | NM |
| Morneault | 1996 | Canada | 1 | F | 0.01 | Blood clots | T, LMB | Suction by bronchoscopy | 62 | 62 | Survival | Survival |
| Isaacson | 1996 | USA | 1 | F | 0.06 | Subglottic stenosis | UT | Tracheostomy | NM | NM | Survival | NM |
| Rosa | 1996 | USA | 1 | F | 53 | Thyroid lymphoma | UT | Tracheostomy | NM | NM | Survival | NM |
| Stewart | 1998 | USA | 1 | M | 19 | Lymphoma | T | Endotracheal intubation | 13 | 17 | Survival | NM |
| Belmont | 1998 | Canada | 1 | F | 73 | Thyroid lymphoma | T | Tracheotomy, biopsy | NM | NM | Survival | Survival |
| Brown | 2003 | UK | 1 | M | 1.17 | Grape aspiration | T | Foreign body extraction | NM | 17 | Survival | Survival |
| Ignacio | 2006 | USA | 1 | F | 1.17 | Bean aspiration | T | Foreign body extraction | NM | 21 | Survival | Survival |
| Smith | 2009 | New Zealand | 2 | M | 46 | Tracheal papillomatosis | T | Mass removal, laser therapy | NM | 3 | Survival | NM |
|  |  |  |  | M | 57 | Tracheal papillomatosis | T | Mass removal, laser therapy | NM | 5 | Survival | NM |
| Cheng | 2009 | USA | 1 | F | 66 | Post-pneumonectomy tracheal and bronchus collapse, stents migration and fracture | T, LMB | Stent removal | NM | NM | Survival | NM |
| Collar | 2010 | USA | 1 | NM | 16 | Recurrent respiratory papillomatosis | C, LMB, RMB | Laser therapy | NM | NM | Survival | NM |
| Willms | 2012 | USA | 1 | M | 39 | Osteogenic sarcoma | LT, C, RMB | Mass removal | NM | NM | Survival | NM |
| Thung | 2012 | USA | 1 | F | 17 | Fibrinous mass, stenosis of bronchial anastomosis site after lung transplant | LMB, RMB | Balloon dilatation, sent placement | NM | NM | Survival | NM |
| Hong | 2012 | Korea | 1 | M | 69 | Gastric cancer | T | Stent placement | NM | NM | Survival | NM |
| Gourdin | 2012 | Belgium | 1 | M | 27 | Post-intubation, post-tracheotomy, airway stent obstructed by granuloma | LT | Stent removal, laser therapy | NM | NM | Survival | NM |
| George | 2012 | USA | 1 | F | 36 | Tracheobronchomalacia, airway stent obstructed by granuloma | T, LMB, RMB | Stent removal and replacement | NM | NM | Survival | NM |
| Hong | 2013 | Korea | 18 | F | 68 | Thyroid cancer | T | Stent placement | NM | NM | Death | Death |
|  |  |  |  | F | 43 | NSCLC | T | Stent placement | NM | NM | Death | Death |
|  |  |  |  | M | 78 | Head and neck cancer | T | Endotracheal intubation | NM | NM | Survival | Survival |
|  |  |  |  | M | 58 | Post-intubation tracheal stenosis | T | Stent removal | NM | NM | Survival | Survival |
|  |  |  |  | F | 43 | NSCLC | T | Mass removal | NM | NM | Survival | Survival |
|  |  |  |  | M# | 75 | SCLC | T | Stent placement | NM | NM | Survival | Survival |
|  |  |  |  | M# | 75 | SCLC, sent migration | T | Stent removal | NM | NM | Death | Death |
|  |  |  |  | M | 60 | NSCLC | T | Mass removal | NM | NM | Survival | Survival |
|  |  |  |  | F | 49 | Adenoid cystic carcinoma | T | Mass removal | NM | NM | Survival | Survival |
|  |  |  |  | M | 82 | Esophageal cancer | T | Stent placement | NM | NM | Survival | Survival |
|  |  |  |  | M | 53 | Renal cell carcinoma | T | Stent placement | NM | NM | Survival | Survival |
|  |  |  |  | F | 67 | Tracheobronchial stenosis | T | Stent placement | NM | NM | Death | Death |
|  |  |  |  | M | 16 | Bronchial cancer | T | Mass removal | NM | NM | Survival | Survival |
|  |  |  |  | M | 19 | Lymphoma | T | Mass removal | NM | NM | Survival | Survival |
|  |  |  |  | M | 65 | NSCLC | T | Mass removal | NM | NM | Survival | Survival |
|  |  |  |  | M | 61 | Thyroid cancer | T | Stent placement | NM | NM | Death | Death |
|  |  |  |  | M | 58 | NSCLC | T | Mass removal | NM | NM | Survival | Survival |
|  |  |  |  | M | 70 | NSCLC | T | Mass removal | NM | NM | Survival | Survival |
|  |  |  |  | M | 70 | Schwannoma | T | Mass removal | NM | NM | Survival | Survival |
| Chang | 2013 | Taiwan | 1 | M | 80 | SCLC | LMB | Photodynamic therapy, mass removal | NM | 26 | Survival | Survival |
| Metcalf | 2013 | USA | 1 | F | 52 | Sand aspiration | LMB | Bronchoalveolar lavage, foreign body extraction, | 33 | NM | Survival | Survival |
| Wang | 2014 | China | 1 | F | 34 | Lymphoma | Esophageal fistula, LT | Stent removal, endotracheal intubation | NM | 28 | Survival | Survival |
| Kim | 2014 | Korea | 1 | F | 88 | Mediastinal teratoma | T | Mass removal, endotracheal intubation | NM | NM | Survival | Survival |
| Park JM | 2014 | Korea | 1 | F | 40 | Airway hemorrhage | RMB | Endobronchial balloon occlusion and hemostatic therapy | NM | 85 | Survival | Survival |
| Park AH | 2014 | Korea | 3 | M | 0.67 | Kidney bean aspiration | RMB | Foreign body extraction | NM | NM | Survival | NM |
|  |  |  |  | M | 1.83 | Almond aspiration | T | Foreign body extraction | NM | NM | Survival | NM |
|  |  |  |  | F | 2 | Peanut aspiration | LMB | Foreign body extraction | NM | NM | Survival | NM |
| Ko | 2015 | Canada | 1 | F | 36 | Squamous cell  carcinoma of trachea | LMB, RMB | Stent placement | NM | 3 | Survival | Survival |
| McLenon | 2016 | USA | 1 | M | 59 | NSCLC | RMB | Stent placement | NM | NM | Survival | NM |
| Natt | 2016 | USA | 1 | F | 53 | Post-tracheostomy stenosis | UT | Balloon dilation, stent plecement | NM | NM | Survival | NM |
| Park JH | 2017 | Korea | 14 | M | 60 | Lung cancer | RMB, LMB | Stent placement | 4 | 17 | Survival | NM |
|  |  |  |  | M | 71 | Tracheal cancer | UT | Stent placement | 6 | 17 | Survival | NM |
|  |  |  |  | F | 50 | Tonsil lymphoma | RMB | Stent placement | 41 | 45 | Survival | NM |
|  |  |  |  | M | 69 | Esophageal cancer | UT | Stent placement | 4 | 5 | Survival | Death |
|  |  |  |  | M | 67 | Thyroid cancer | UT | Stent placement | 3 | 4 | Survival | NM |
|  |  |  |  | M | 30 | Lung cancer | UT | Stent placement | 10 | 23 | Survival | NM |
|  |  |  |  | F | 56 | Lung cancer | LMB | Stent placement | 6 | 11 | Survival | NM |
|  |  |  |  | F | 59 | Lung cancer | LT, C | Stent placement | 3 | 6 | Survival | NM |
|  |  |  |  | M | 57 | Thymus cancer | LT, C | Stent placement | 3 | 7 | Death | Death |
|  |  |  |  | M | 66 | Lung cancer | LT, C | Stent placement | 12 | 32 | Death | Death |
|  |  |  |  | M | 82 | Lung cancer | LT, C | Stent placement | 2 | 13 | Survival | NM |
|  |  |  |  | M | 61 | Lung cancer | LMB | Stent placement | 3 | 18 | Survival | NM |
|  |  |  |  | M | 77 | Lung cancer | LT, RMB | Stent placement | 10 | 28 | Survival | NM |
|  |  |  |  | M | 41 | Post-tracheostomy stenosis | UT | Stent placement | 11 | 20 | Survival | NM |
| Fung | 2017 | Australia | 1 | F | 73 | Sinonasal melanoma | LT | Mass removal | NM | NM | Survival | Survival |
| Ramírez-Romero | 2017 | Spain | 1 | F | 15 | Inflammatory bowel disease | T | Mass removal | NM | NM | Survival | NM |
| Nokes | 2018 | USA | 1 | F | 49 | Myeloma | LMB, RMB | Stent placement | NM | NM | Survival | Survival |
| Vobruba | 2018 | Czech | 1 | F | NM | Pulmonary agenesis | LMB, RMB | Stent placement | NM | NM | Survival | Death |
| Yamada | 2018 | Japan | 1 | F | 67 | Inflammation of bronchial tissue | T | Biopsy, endotracheal intubation | NM | NM | Survival | NM |
| Yunoki | 2018 | Japan | 6 | M | 67 | NSCLC | LMB | Tracheostomy, endotracheal intubation | NM | 38 | Survival | Survival |
|  |  |  |  | M | 53 | NSCLC | RMB | Endotracheal intubation | NM | NM | Survival | Death |
|  |  |  |  | M | 63 | Tracheal stenosis | UT | Tracheostomy | NM | 18 | Survival | Survival |
|  |  |  |  | F | 85 | Adenomatous goiter | T | Endotracheal intubation | NM | NM | Survival | Survival |
|  |  |  |  | F | 60 | Lymphoma | T | Endotracheal intubation | NM | NM | Survival | Survival |
|  |  |  |  | F | 74 | NSCLC | LT, RMB | Tracheostomy, endotracheal intubation | NM | NM | Death | Death |
| Munakata | 2020 | Japan | 1 | F | 47 | Blood clots | LT | Blood clots removal | NM | NM | Death | Death |
| Pu | 2020 | China | 7 | M | 65 | Lung cancer | RMB, LMB | Biopsy, mass removal | 2 | 20 | Survival | NM |
|  |  |  |  | M | 60 | Esophageal cancer | UT | Biopsy, mass removal | 3 | 11 | Survival | NM |
|  |  |  |  | M | 51 | Esophageal cancer | UT | Stent placement | 15 | 39 | Survival | NM |
|  |  |  |  | M | 52 | Esophageal cancer | UT | Stent placement | 12 | 12 | Survival | NM |
|  |  |  |  | F | 75 | Cervical Lymphoma | LT,C | Stent placement | 14 | 18 | Survival | NM |
|  |  |  |  | M | 47 | Lung cancer | RMB, C | Biopsy, mass removal | 1 | 15 | Survival | NM |
|  |  |  |  | M | 60 | Lung cancer | RMB,C | Biopsy, mass removal | 12 | 12 | Survival | NM |
| Yu | 2020 | China | 1 | NM | 58 | Esophageal squamous cell carcinoma | LT, C, RMB, LMB | Bronchoscopy, cryotherapy | NM | 3 | Survival | NM |
| Ni Fhlatharta | 2020 | Ireland | 3 | M | 74 | Malignant melanoma | LT | Stent placement | NM | NM | Survival | NM |
|  |  |  |  | M | 65 | Tracheomalacia | UT | Stent placement | NM | NM | Survival | NM |
|  |  |  |  | M | 56 | Neurofibromatosis type 1, tracheomalacia | T | Stent placement | NM | NM | Survival | NM |
| Kitazawa | 2020 | Japan | 1 | F | 39 | Lung adenocarcinoma | T, C, LMB, RMB | Mass removal, stent placement | 15 | NM | Survival | Survival |
| Kuroda | 2020 | Japan | 1 | F | 75 | Thoracic aortic aneurysm | T | Stent placement | NM | NM | Survival | NM |
| Odigwe | 2020 | USA | 1 | F | 30 | Post-tracheostomy tracheal stenosis | UT | Balloon dilatation, endotracheal intubation | NM | NM | Survival | NM |
| Zimmermann | 2020 | France | 1 | M | 83 | Chondrosarcoma | T | Mass removal | NM | NM | Survival | Survival |
| Meyer | 2021 | Belgium | 14 | M | 29 | Post-intubation tracheal stenosis | T | Stent removal and replacement | NM | *7.2 | Survival | Survival |
|  |  |  |  | M | 39 | Post-intubation tracheal stenosis | T | Stent removal and replacement | NM | *7.2 | Survival | Survival |
|  |  |  |  | M | 55 | Post-intubation tracheal stenosis | T | Stent removal and replacement | NM | *7.2 | Survival | Survival |
|  |  |  |  | M | 56 | Post-intubation tracheal stenosis | T | Stent removal and replacement | NM | *7.2 | Survival | Survival |
|  |  |  |  | M | 68 | Postintubation tracheal stenosis | T | Sent replacement | NM | *7.2 | Survival | Survival |
|  |  |  |  | F | 54 | Post-intubation tracheal stenosis | T | Stent removal | NM | *7.2 | Survival | Survival |
|  |  |  |  | M | 60 | Post-intubation tracheal stenosis | T | Stent removal | NM | *7.2 | Survival | Survival |
|  |  |  |  | M | 49 | Post-intubation tracheal stenosis | T | Stent placement | NM | *7.2 | Survival | Survival |
|  |  |  |  | M | 78 | Thyroid carcinoma | T | Stent placement | NM | *7.2 | Survival | Death |
|  |  |  |  | M | 38 | Esticular cancer | LMB, RMB | Stent placement | NM | *7.2 | Survival | Death |
|  |  |  |  | F | 49 | Lung cancer | T, LMB, RMB | Stent replacement | NM | *7.2 | Survival | Death |
|  |  |  |  | M | 59 | Post-pneumonectomy tracheobronchial stenosis | T, LMB, RMB | Stent removal and replacement | NM | *7.2 | Survival | Survival |
|  |  |  |  | M | 59 | Post-pneumonectomy  tracheobronchial stenosis | T, LMB, RMB | Stent removal and replacement | NM | *7.2 | Survival | Death |
|  |  |  |  | M | 62 | Stenosis post lung transplant | LMB, RMB | Stent removal and replacement | NM | *7.2 | Survival | Survival |
| Shirasaki | 2021 | Japan | 1 | M | 24 | Congenital tracheal stenosis | T, C | Balloon dilatation | NM | NM | Survival | NM |
| Siddiqi | 2021 | Japan | 1 | NM | NM | Tracheal malignancy | T | Stent removal and replacement | NM | NM | Survival | NM |
| Watanabe | 2021 | Japan | 1 | F | 66 | Blood clots | T, LMB, RMB | Blood clots removal, endobronchial lavage | 21 | 23 | Survival | Survival |
| Zhang | 2021 | China | 1 | F | 51 | Relapsing polychondritis | T | Stent placement | 9 | NM | Survival | Survival |
| Kawakado | 2022 | Japan | 1 | F | 62 | SCLC | T | Stent placement | NM | NM | Survival | Survival |
| Giani | 2022 | Italy | 1 | M | 47 | Tracheal lesion | T | Stent placement, endotracheal intubation | NM | NM | Survival | Survival |

Supplementary table 1. Characteristic of patients with critical airway problems undergoing ECMO and interventional therapy.

*data represent mean of the included studies; #, one patient underwent VV ECMO twice; NM, not mentioned; ECMO: extracorporeal membrane oxygenation; NSCLC, non-small cell lung cancer; SCLC, small cell lung cancer; T, tracheal; UT, upper tracheal; LT, lower tracheal; C, carina; LMB, left main bronchial; RMB, right main bronchial; M, male; F, female; No., number.
