# Supplementary material for: Extracorporeal membrane oxygenation in critical airway interventional therapy: A review
Source: Front Oncol. 2023 Mar 27;13:1098594. doi: 10.3389/fonc.2023.1098594 (PMC10083385; doi:10.3389/fonc.2023.1098594)
Supplement: Supplementary file 2 [file Table_2.docx]

| Study | No. | ECMO initiation | ECMO mode | Cannulation | Anticoagulation | Coagulation index | Target range, seconds | ECMO duration, hours | Weaning off ECMO | ECMO related complications |
| --- | --- | --- | --- | --- | --- | --- | --- | --- | --- | --- |
| Higashi 1989 | 1 | Emergent | VV | NM | NM | NM | NM | 36 | Yes | None |
| Morneault 1996 | 1 | Emergent | VV | IJV | UFH | ACT | 160-180 | 208 | Yes | None |
| Isaacson 1996 | 1 | Emergent | VV | IJV | NM | NM | NM | NM | Yes | None |
| Rosa 1996 | 1 | Emergent | VA | FFVA | NM | NM | NM | NM | Yes | None |
| Stewart 1998 | 1 | Emergent | VA | FFVA | UFH | ACT | 180-200 | 48 | Yes | Foot ischemia |
| Belmont 1998 | 1 | Emergent | VA | FFVA | UFH | NM | NM | NM | Yes | None |
| Brown 2003 | 1 | Emergent | VA | NM | NM | NM | NM | 144 | Yes | None |
| Ignacio 2006 | 1 | Emergent | VA | NM | UFH | ACT | 200-220 | 72 | Yes | None |
| Smith 2009 | 2 | Emergent | VV (2) | FJV (2) | UFH (2) | ACT | 190-250,  140-210 | NM | Yes | One case had neuropraxia of cannulation site |
| Cheng 2009 | 1 | Emergent | VA | NM | NM | NM | NM | NM | Yes | None |
| Collar 2010 | 1 | Emergent | VV | FJV | UFH | NM | NM | NM | Yes | None |
| Willms 2012 | 1 | Emergent | VA | FFVA | UFH | ACT | 180-200 | NM | Yes | None |
| Thung 2012 | 1 | Emergent | VV | NM | NM | NM | NM | NM | Yes | None |
| Hong 2012 | 1 | Preventive | VV | FJV | UFH | ACT | 180-200 | 2 | Yes | None |
| Gourdin 2012 | 1 | Preventive | VV | FJV | UFH | ACT | 180 | NM | Yes | None |
| George 2012 | 1 | Preventive | VV | BFV | UFH | NM | NM | NM | Yes | None |
| Hong 2013* | 18 | Emergent | VV (19) | BFV (19) | UFH+nafamostat mesilate (6)  UFH (13) | ACT (19) | 130-150 | 26.4# | Yes | One case had massive airway bleeding. |
| Chang 2013 | 1 | Emergent | VA | NM | NM | NM | NM | 96 | Yes | None |
| Metcalf 2013 | 1 | Emergent | VA | FFVA | NM | NM | NM | NM | Yes | None |
| Wang 2014 | 1 | Emergent | VA | FFVA | UFH | NM | NM | NM | Yes | None |
| Kim 2014 | 1 | Preventive | VV | BFV | UFH | NM | NM | 2.3 | Yes | None |
| Park JM 2014 | 1 | Emergent | VV | NM | None | NM | NM | 264 | Yes | None |
| Park AH 2014 | 3 | Emergent | VV (2)  VA (1) | NM (2)  IJVA (1) | NM | NM | NM | NM | Yes | None |
| Ko 2015 | 1 | Emergent | VV | IJV | UFH | ACT | 200 | NM | Yes | None |
| McLenon 2016 | 1 | Preventive | VV | BFV | NM | NM | NM | 48 | Yes | None |
| Natt 2016 | 1 | Emergent | VV | NM | NM | NM | NM | NM | Yes | None |
| Park JM 2017 | 14 | Emergent (3)  Preventive (11) | VV (14) | FJV (3)  BFV (11) | UFH+nafamostat mesilate (6)  UFH (8) | ACT | 130-150 | 42# | Yes | One case had right femoral vein rupture and hematoma, one case had fistula  between the superior femoral artery and the adjacent femoral vein |
| Fung 2017 | 1 | Preventive | VV | BFV | None | NM | NM | NM | Yes | None |
| Ramírez-Romero 2017 | 1 | Emergent | VV | NM | NM | NM | NM | NM | Yes | None |
| Nokes 2018 | 1 | Preventive | VV | BFV | NM | NM | NM | NM | Yes | None |
| Vobruba 2018 | 1 | Emergent | VV | NM | NM | NM | NM | 432 | Yes | None |
| Yamada 2018 | 1 | Emergent | VV | FJV | UFH | NM | NM | NM | Yes | None |
| Yunoki 2018 | 6 | Emergent (1)  Preventive (5) | VV (6) | BFV (4)  FJV (2) | UFH (6) | ACT | 250 | 17.7# | Yes | None |
| Munakata 2020 | 1 | Emergent | VA | NM | NM | NM | NM | NM | No | None |
| Pu 2020 | 7 | Preventive | VV (7) | FJV (7) | NM | NM | NM | 34# | Yes | None |
| Yu 2020 | 1 | Preventive | VV | FJV | UFH | ACT | 250 | NM | Yes | Airway bleeding |
| Ni Fhlatharta  2020 | 3 | Preventive | VV (3) | NM | NM | NM | NM | NM | Yes | None |
| Kitazawa 2020 | 1 | Emergent | VV | NM | NM | NM | NM | 24 | Yes | None |
| Kuroda 2020 | 1 | Emergent | VV | FJV | NM | NM | NM | NM | Yes | None |
| Odigwe 2020 | 1 | Emergent | VV | NM | NM | NM | NM | NM | Yes | None |
| Zimmermann 2020 | 1 | Preventive | VV | BFV | UFH | NM | NM | NM | Yes | None |
| Meyer 2021 | 14 | Preventive | VV (8)  VA (6) | FJV (8)  FFVA (6) | UFH (14) | ACT | 180-200 | 129# | Yes | Four cases airway bleeding |
| Shirasaki 2021 | 1 | Emergent | VV | NM | NM | NM | NM | 72 | Yes | None |
| Siddiqi 2021 | 1 | Preventive | VV | FJV | NM | NM | NM | NM | Yes | None |
| Watanabe 2021 | 1 | Emergent | VV | NM | UFH | ACT | 180-200 | 100 | Yes | Airway bleeding |
| Zhang 2021 | 1 | Emergent | VV | FJV | NM | NM | NM | 216 | Yes | None |
| Kawakado 2022 | 1 | Emergent | VV | NM | NM | NM | NM | 1.7 | Yes | None |
| Giani 2022 | 1 | Emergent | VV | IJV | NM | NM | NM | 96 | Yes | None |

Supplementary table 2. ECMO variables of patients with critical airway problems undergoing ECMO and interventional therapy.

*, one patient underwent VV ECMO twice; #, data represent median; VA: venoarterial; VV: venovenous; ECMO: extracorporeal membrane oxygenation; BFV, both femoral vein; FJV, femoral and internal jugular vein; FFVA, femoral vein and artery; IJV, single cannulation with a double lumen in the internal jugular vein; IJVA, internal jugular vein and the arterial cannula; NM, not mentioned; No., number; ACT: activated clotting time; UFH: unfractionated heparin.
